# Broken Bones

**Published:** 1859-02

**Authors:** 


### BROKEN BONES

May be prevented in icy weather by taking steps short and slow, but fast and long in all weathers, in a direction from a mad bull.

If, by a neglect of these reasonable precautions, a bone is broken, the first thing to be done is to groan with an earnestness prodigious; don't yell, for that repels the hearer, while the former attracts by sympathy. Besides, groans, like tears, bring relief. Tearless silence is the sad precursor of certain death in all great bodily ailments.

Persons have added to their injuries before now by attempting to rise, and falling down again, in consequence of a limb having been broken. This may be avoided, if, on the first return to consciousness, after a " collision," bursting of a boiler, and the like, a man would take the precaution, or have the presence of mind, before attempting to rise, to endeavor to move each leg and arm; for, if he can, neither is broken, nor are any of their joints dislocated; upon obtaining which intelligence there can be no rational obstacle to the most expeditious pedestrianism which the emergencies of the case admit of.

